# General Practitioners' Participation in a Large, Multicountry Combined General Practitioner-Patient Survey: Recruitment Procedures and Participation Rate

**DOI:** 10.1155/2016/4929432

**Published:** 2016-03-07

**Authors:** Peter P. Groenewegen, Stefan Greß, Willemijn Schäfer

**Affiliations:** ^1^Netherlands Institute for Health Services Research (NIVEL), P.O. Box 1568, 3500 BN Utrecht, Netherlands; ^2^Department of Sociology and Department of Human Geography, Utrecht University, Heidelberglaan 2, 3584 CS Utrecht, Netherlands; ^3^Hochschule Fulda University of Applied Sciences, Leipziger Straße 123, 36037 Fulda, Germany

## Abstract

*Background.* The participation of general practitioners (GPs) is essential in research on the performance of primary care. This paper describes the implementation of a large, multicountry study in primary care that combines a survey among GPs and a linked survey among patients that visited their practice (the QUALICOPC study). The aim is to describe the recruitment procedure and explore differences between countries in the participation rate of the GPs.* Methods.* Descriptive analyses were used to document recruitment procedures and to assess hypotheses potentially explaining variation in participation rates between countries.* Results.* The survey was implemented in 31 European countries. GPs were mainly selected through random sampling. The actual implementation of the study differed between countries. The median participation rate was 30%. Both material (such as the payment system of GPs in a country) and immaterial influences (such as estimated survey pressure) are related to differences between countries.* Conclusion.* This study shows that the participation of GPs may indeed be influenced by the context of the country. The implementation of complex data collection is difficult to realize in a completely uniform way. Procedures have to be tuned to the context of the country.

## 1. Background

The participation of general practitioners (GPs) is essential in research on the performance of primary care. Rates of participation are not always satisfactory. This paper describes and analyses the rate of participation of GPs in a large multicountry study, the QUALICOPC (Quality and Costs of Primary Care) study.

The study consists of linked surveys among GPs and their patients, with the patient survey implemented through field workers that visited the practices, in 31 European countries between 2011 and 2013. Recruitment of GPs to participate was challenging and rates of participation varied per country and were often relatively low. A brief summary of the study design of the QUALICOPC study is provided in Box 1. This paper describes the recruitment procedures and the rates of participation of GPs, providing the background information to publications based on the QUALICOPC study and to future users of the data or researchers who want to organize a comparable survey. We also analyse explanations for the varying participation rates to contribute to our knowledge on the implementation of complex international field studies.

As in any study of nonresponse, willingness to participate will vary between individuals. However, in multicountry studies there might also be systematic variation related to influences that are the same for all GPs in a country. This variation might be related to the way the study is implemented, but also to position of GPs. Consequently, response may be selective within each country, but also between countries.

Studies that review survey participation rates of GPs usually review single survey, single country studies. These studies may be relevant for comparison, but only to a limited extent. Nevertheless, they may be helpful in developing ideas about between country variation. Recent review studies of health care worker response rates [1] or GP response rates [2] show that monetary and nonmonetary incentives increase response, as do prior contact by telephone and email and reminders. A systematic review [1] also reports on differences between countries, with European countries showing higher response rates compared to the US, Canada, and Australia/New Zealand. However, they focus on explaining differences by characteristics of the surveys, leaving country context out.

Our research questions are as follows:

1. * How was recruitment of GPs in the QUALICOPC study organized and what is the resulting participation rate per country?*
2. * How does the willingness of GPs to participate in this combined GP and patient survey vary between countries and how can this variation be explained?*

We hypothesize that differences in participation rate between countries are related to material and immaterial influences [6] and that these may be dependent on the implementation of the study and on country context.

### 1.1. Material Influences

(H1) Participation in a study, such as QUALICOPC, takes time and effort that could also be used in other activities. Hence, there are opportunity costs that may vary systematically between countries. If the opportunity costs for time spent in participating are higher, we expect less GPs to do it. The implication is that in countries where GPs earn more they might be less willing to participate (country context).
(H2) In countries where GPs are paid fee for service, the opportunity costs of participation are more salient and they might be more used to receive material incentives. We expect lower participation among GPs in these countries and more participation in countries where GPs are salaried (country context).
(H3) In countries where GPs were given a material incentive for participation in the study, we expect a higher willingness to participate (study implementation).

### 1.2. Immaterial Influences

Immaterial influences could work via several mechanisms: survey fatigue [7], prosocial (or altruistic) attitudes [8], and contribution to a collective good [9].

(H4) The number of requests to participate in surveys might differ between countries and lead to “survey fatigue.” Willingness to participate will be lower in countries with a large survey load, assuming that GPs have a bigger chance to have been invited to participate in surveys in the past (country context).
(H5) By participating in our study GPs do others (the researchers or the country coordinators that have approached them) a favour. GPs may be more inclined to do so if they have prosocial attitudes. Countries differ in the prevalence of these attitudes. Hence, if GPs live in a country with in general more prosocial attitudes, we expect them to be more willing to participate (country context).
(H6) Participation in our study contributes to a collective good, by providing information about the position of GPs that may be used by, for example, professional organizations to plea for better conditions for primary care. We hypothesize that GPs feel more induced to contribute to the collective good when their numbers are smaller, when their structural position is weaker (country context), and when an appeal has been done by their professional organization to participate (study implementation).

## 2. Methods

### 2.1. Recruitment Procedures and Participation Rate

The intended recruitment procedures have been described elsewhere [4]. We briefly summarize them here. In Section 3 we will describe the actual implementation.

Data were to be collected in 31 European countries and three non-European countries. This paper is limited to a description of the European countries. Descriptions of the recruitment in Australia and Canada are described elsewhere [10, 11]. The target response was 220 GPs per country, except for the four smallest countries where the target was 75. In each country we aimed to draw a nationally representative sample of GPs with one GP per practice. In countries with no national register, multistage sampling was to be used, for example, by combining registers from different regions. Furthermore, in large countries with regional differences in health care system, several representative regions would be selected and subsequently GPs within these regions. The information on the actual implementation of the study was provided by the national coordinators. Based on their information on the numbers of GPs invited and the actual number in the database we calculated the participation rate.

Ethical approval for the QUALICOPC study was acquired in accordance with the legal requirements in each country.

### 2.2. Data to Assess the Hypotheses

To assess the hypotheses, data were derived from several sources:

- GP income: estimates from the PHAMEU database [3, 12].
- Material incentive: see Table 1.
- GP payment system: fee-for-service, mixed systems of capitation and fee-for-service, or salary, PHAMEU database [3].
- Survey pressure: indicated by the number of abstracts reporting on GP surveys at WONCA Europe conferences from 2008 to 2012, grouped in 5 categories (additional file 1, in Supplementary Material available online at http://dx.doi.org/10.1155/2016/4929432). Abstracts have a lower threshold than published research papers. As the conferences are always in a different place in Europe, the bias towards Northwest Europe is less strong.
- Prosocial attitudes: percentage of the general population saying that most people can be trusted. Most recent data are available per country (oldest 1990–94, 2 countries, most recent 2010–2014, 10 countries), 9 countries missing [13].
- Number of GPs per country: calculated from GPs per 100.000 population [3] and country population [14].
- Structural position of GPs: overall strength of the structure of primary care [3, 15].
- Appeal by professional organizations: letter of recommendation used in recruitment (see Table 1).

Apart from the variables in the hypotheses we have included two confounders: total population size and gross domestic product (as deviation from the EU28) [14].

### 2.3. Analyses

Due to the small number of countries we have analysed the data pragmatically in order to assess the hypotheses. We have grouped the countries according to participation rate into three groups: participation rates low (≤20%), medium (21–50%), and high (>50%). As a sensitivity test we have also used alternative cut-off points (≤30%, 31–60%, and >60%). For each of the groups we have computed the average or percentage of the explanatory variables from the hypotheses and the two confounders.

## 3. Results

### 3.1. Implementation of the Study and Participation Rates

The aim was to include at least all EU member states. However, for different reasons the study could not be implemented in France.

We have categorized the actual sampling procedure. In four small countries the (almost) entire population of GPs was invited to participate (A in Table 1). Random sampling was used in the majority of countries, either from a national sample (17 countries, B) or from preselected regions (4 countries, C). In four countries random sampling was complemented with opportunity sampling (D) and in two countries opportunity sampling [16] was used (E). In most countries selected GPs were contacted through a mix of letters, email, and telephone contact. One or more reminders were sent to the selected GPs. The implementation of the patient survey was in most cases done through field workers who visited the practice to invite patients to fill in the patient questionnaire (with the exception of Denmark and England and a part of Norway and Sweden). Approximately half of the countries used a material incentive, but in widely different form and size. Just under half of the countries used a support letter from a professional organization. The median duration of the data collection, including the patient survey, was half a year.

Table 2 gives the estimated participation rates. These vary from less than 10% in Austria, Belgium, Germany, Ireland, and Sweden to over 70% in Cyprus, Greece, Iceland, Malta, and Spain.

For most countries we could check the representativeness of the participating GPs by comparing them with regard to age and gender to national statistics (additional file 2) [17]. Average age of the participating GPs differed from the national average by five years or more in Hungary, Italy, and Spain. The percentage of female GPs in our study differs by ten percentage points or more in Greece, FYR Macedonia, the Netherlands, Slovenia, and Spain.

### 3.2. Assessing the Hypotheses

Table 3 contains the information to evaluate the hypotheses.

Average GP income is highest in the low response group. There is no trend over all three groups. This partly confirms hypothesis 1.

A material incentive was more often provided in the low response group and less often in the medium and high response groups, opposite to the relationship of hypothesis 2.

In the higher response groups the percentage of countries with predominantly salaried GPs is higher. This confirms our hypothesis that opportunity costs are related to participation. However, there is no trend for the percentage of countries with predominant pure fee-for-service or mixed payment systems. Therefore the hypothesis is partly confirmed.

Survey load is lowest in the high response group, but there is no trend over all three groups, which partly confirms hypothesis 4.

The percentage of people that say most people can be trusted is lower in higher response groups. Hypothesis 5 was therefore refuted.

For hypothesis 6, we used three indicators. In the low response group the average number of GPs per country is higher, but there is no trend over all groups, which partly confirms the hypothesis. GPs in countries with a weaker primary care system seem to be more inclined to participate than those in countries with stronger primary care. In contrast with the hypothesis, the share of countries using a support letter is lowest in the high response group and highest in the low response group. In summary, hypothesis 6 is partly confirmed.

As far as the two confounders are concerned, we see a trend towards smaller countries and countries with lower GDP per capita in the higher response groups.

## 4. Discussion

The actual implementation of the QUALICOPC study differs somewhat from the intended approach. The country coordinators received uniform instructions but also had to take into account the feasibility of the suggested procedures in the context of their own country. Moreover, the financial resources for the implementation of the study in each country were modest. Consequently, the country coordinators had to use their creativity to come as close as possible to the suggested procedures within the restrictions of time, money, and national circumstances. In some countries there has been a stepwise deviation from the original instructions when it turned out that these were not resulting in the expected participation.

A large number of European GPs have participated in the study with a multiple of patients. In half of the countries a participation rate among GPs of over 30% was realized. Against the background of decreasing participation rates of physicians in surveys and large differences between countries [1], this is satisfactory. Random sampling of GPs was realized in two-thirds of countries. Only in France we did not succeed in implementing the study. We have, after several attempts, not been able to locate a team that was willing and able to take up the national coordination for data collection in that country, within the requested time and financial frame.

Differences in procedures and in participation rates between countries might have led to unknown levels of bias. We have attempted to estimate bias by comparing the samples of GPs to national data on age and gender of the population of GPs. The participating GPs are by and large representative on age and gender for the population of GPs in their country. Nevertheless, the low response and the efforts to reach the target suggest self-selection, probably in the direction of GPs who are interested in research and developing their profession.

An important reason for low response was probably that participation involved allowing field workers to come to the practice to ask patients to fill in a questionnaire. GPs may have many reasons not to want this [18]. They may see it as a disruption of practice routines, expect question from their patients, and so forth. Moreover, the combination with a patient survey changes the context of the survey from individual GPs to the practice. Filling in a questionnaire by a GP is an individual decision but becomes a practice decision when patients are also involved and field workers visit the practice. Primary care practices are organizations (although often small) and, in general, organizations behave differently from individuals. They tend to follow procedures to decide about requests, with as a result a longer procedure and lower chance of spontaneous participation compared to individuals [19–22]. GP participation is also related to the role of other staff that have to pass a request or a questionnaire on to the GP [10, 23]. In sum, limitations for the QUALICOPC study are the differences in implementation between countries and low participation rate of GPs.

The QUALICOPC study includes enough countries to analyse differences in participation rates between countries. It was hypothesized that material and immaterial influences would affect the decision to participate. The participation rate was indeed related to material factors, but not always in the hypothesized direction. The response rate to the QUALICOPC study was low in countries with higher average income of GPs and in countries where a material incentive was more often used. The latter might be a case of reverse causation in that coordinators might have proposed material incentives because of their estimation that participation rates are low in their country. Moreover, in some countries it might have become common practice to give a material incentive for research participation, perhaps as a reaction to decreasing participation rates. However, we have not found information in the literature on such practices. Participation was high in countries with predominantly salaried GPs.

The response rate was also found to be related to immaterial factors. In the high response group survey load was lower. Prosocial attitudes were related to response rates but in the opposite direction of the hypothesis. We only had information for a selection of countries and prosocial attitudes were measured in the population and not amongst GPs. The propensity to participate as a contribution to the collective good was analysed through the number of GPs, the strength of primary care, and the support of a professional organization. For support letters this hypothesis was refuted and for the two other operationalizations it was partly confirmed. The collective good mechanism is usually connected to much smaller numbers than we find with the number of GPs in a country. However, the professions seem to be able to produce collective goods in the situation of much larger numbers of coproducers [24, 25]. As concerns the role of strong primary care, it could also be reasoned that in countries with stronger primary care systems GPs are more aware of their leading position internationally and want to confirm that by participating in international research. However, this alternative line of reasoning was not confirmed by our analysis.

A limitation of the test of our hypotheses is that some country coordinators have introduced material and immaterial incentives in the course of recruitment in response to disappointing participation. Consequently, the effects of incentives might be underestimated.

An alternative grouping of the countries according to participation rates, as a sensitivity analysis, largely gave the same results, but for hypothesis 2 there was a less clear gradient with the percentage with a material incentive nearly the same in the low and middle response categories; also for hypothesis 3 there was a less clear gradient with the lowest percentage salaried GPs in the middle response category.

Although we were able to include a large number of countries in this study, from a purely statistical point of view it still is a limitation that the number of countries is too small to do multivariable analysis. As the independent variables are correlated, we are not able to determine which is the most important. The response pattern could also be influenced by other unmeasured variables or confounders. We have looked at two confounders and they differ between the response groups. However, the number of countries is too small to assess the hypothesized effects while taking confounders into account. Participation was higher in smaller and less wealthy countries. However, we prefer the interpretation in terms of our hypotheses over an interpretation in terms of variables that have no specified mechanism.

For the design of future international studies on general practice with comparable size and complexity, we think the following issues are important. The first one is slightly trivial, but important: a comparable study in the future should be able to allocate more money for the implementation of the study. Secondly, the role of national coordinators is of prime importance, because the knowledge of the national situation is indispensable. Thirdly, piloting of the fieldwork procedures should be done in all countries involved. Finally, it might be considered to subcontract the actual fieldwork in a country to a specialized organization, under the direct supervision of the national coordinator; this could improve the uniformity of the fieldwork, although subcontracting is known to bring its own problems often. Our analysis confirmed a lack of strong evidence about the role of material and immaterial incentives in increasing the willingness of GPs to participates. The current systematic reviews on these subjects do not adequately take the country context into account. However, we hope that future reviews of participation rates and response rates will specifically analyse country or health care system differences.

## 5. Conclusion

This paper adds to the literature on survey participation by GPs by bringing in the international comparative perspective. Existing reviews do not address the country context. This study shows that country context has an influence on participation rates. The implication from the QUALICOPC study is that a uniform approach of data collection across countries is almost impossible to achieve. Procedures have to be tuned to the context of the country and available financial resources.

## Supplementary Material

Additional file 1: A measure of survey pressure was derived from the abstracts submitted to Wonca Europe for its yearly conference in 2008 through 2012.
Additional file 2: To assess the representativeness of the responding GPs per country, information on background characteristics of the population of GPs was collected.

## Figures and Tables

**Box 1:**
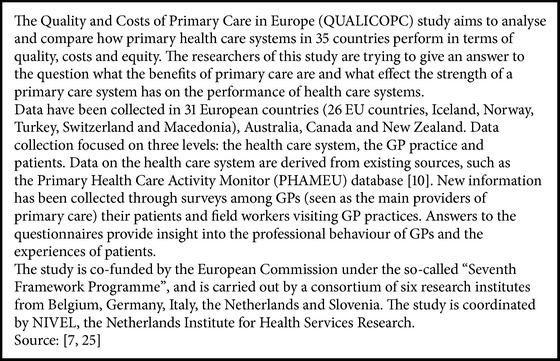
**Box 1: **Study design of the QUALICOPC study.

**Table 1:** Recruitment procedures.

Country	Sampling procedure^*∗*^	Recruitment methods	Reminders^*∗∗*^	Incentives	Fieldworkers	Supporting bodies	Duration survey^*∗∗∗*^
Austria	B	E-mail, personal	4 to 5	€90	Yes	Austrian Society of General Practitioners (ÖGAM), GP education networks of Medical Universities in Austria, GP research network of the Medical University of Vienna, district GPs	9 months

Belgium	B	Letter, telephone, e-mail	Not known	Partly: €30 (voucher, cash for the practice or donated to charity) Partly: nothing	Yes	National Institute for Health and Disability Insurance (RIZIV/INAMI)	23 months

Bulgaria	B	Telephone, face-to-face	2	None	Yes	None	2 months

Cyprus	A	Letter, telephone	Not known	A thank you letter and a small gift (pen)	Yes	Ministry of Health	3 months

Czech Republic	B	Letter, telephone, personal contact	Not known	A textbook on General Practice	Yes	Czech Society of General Practice, Czech Medical Society Jan Evangelista Purkyne, First Faculty of Medicine, Charles University in Prague, Czech Health Statistics Office	5.5 months

Denmark	B	E-mail	2	€98,67	No	The Danish College of General Practitioners	4 months

England	C	Letter and E-mail	1 or more	£100	No	The National Institute for Health Research Primary Care Research Network	8 months

Estonia	A	Letter, telephone, e-mail	2	Credits for personal certification and accreditation of practice (based on the certificate issued by the University of Tartu)	Yes	Estonian Association of Family Doctors	5.5 months

Finland	D	Letter, e-mail, telephone, personal contact	Not known	None	Yes	General practitioners in Finland (GPF) (union of doctors in health centres) and the Finnish Association for General Practice	12 months

Germany	B	Letter	Maximum 1	Lottery 1 Apple iPad	Yes	German College of General Practitioners and Family Physicians (DEGAM) (support was mentioned, but no letter from the professional organization)	11 months

Greece	D	Telephone, letter	Not known	Certificate for their participation and also a gratitude letter on behalf of the Scientific Coordinator	Yes	The Greek Association of General Practitioners (ELEGEIA)	11 months

Hungary	D	E-mail and personal contact	Not known	Small financial opportunity for choosing books from a list, free-educational courses	Yes	All the other 3 departments of family medicine	7 months

Iceland	A	Letter and personal contact	None	An invitation to participate in a seminar were the Icelandic data will be presented	Yes	Icelandic Family Physician Association; Directorate of Health and Ministry of Welfare	3 months

Ireland	D	Letter, e-mail, personal contact, advertisement	Not known	10 × €500 prices in draw and possibility of obtaining internal credits for professional competence requirements	Yes	The Irish College of General Practitioners (ICGP) The ICGP Research Committee and Research Network	11 months

Italy	E	Telephone	Not known	None	Yes	World Organization of Family Doctors (WONCA) Italia	15 months

Latvia	B	Telephone, e-mail	2 to 3	Credit points	Yes	Riga Stradins University. Rural Family doctors association of Latvia	3 months

Lithuania	B	Personally	Not known	None	Yes	None	3.5 months

Luxembourg	A	Telephone	None	None	Yes	Clinical and Epidemiological Investigation Center (CIEC), Luxemburg Institute of Health	9.5 months

Macedonia	B	Letter and e-mail	6-7	None	Yes	Macedonian Association of GP/FM specialists	2.5 months

Malta	B	Telephone	None	Financial reimbursement of €80	Yes	Malta College of Family Doctors The Foundation Programme Malta	3 months

Netherlands	B	Letter, e-mail, telephone	3 to 5	5 GPs could win an iPad	Yes	None	14.5 months

Norway	E	Letter, e-mail, telephone	1 to 4	Gift cards	Yes, partially	The four General Practice Research Units in Norway	6 months

Poland	C	Letter, telephone, e-mail	None	Books (literature & fiction)	Yes	Regional branches of the College of Family Physicians	6.5 months

Portugal	B	Letter, e-mail, telephone	2 to 3	Certificate	Yes	Management Boards of the five Health Regions, the National GP Association and the National GP newspaper	5 months

Romania	B	Letter, telephone, e-mail, personal contact	1	None	Yes	The Romanian College of Physicians	2.5 months

Spain	C	E-mail, telephone	Not known	CME points	Yes	(i) Research Units of Primary Care (ii) Primary Health Centres accredited to train GPs (Training Units for GPs) (iii) Institute of Research of Primary Care of Catalonia	8 months

Slovakia	B	Letter, telephone, personal contact	Maximum 5	Lottery: first tombola price is a 4-day trip to Amsterdam and Utrecht for 2 persons. Prices 2. to 50. are different small practical presents for surgery use, f.e. Tonometers, glucometers, medical books, workpads, and so forth	Yes	Slovak Society of General Practice	5 months

Slovenia	B	Letter, telephone, e-mail	2	None	Yes	Institute for Development of Family Medicine, Medical Chamber	11 months

Sweden	B	Letter	1	None	Yes, partially	Swedish Medical Association, General Practice division and their Research Committee	7.5 months

Switzerland	B	Letter, telephone	Maximum 1	A book voucher (€81)	Yes	Two main medical associations (family medicine and General Internal Medicine)	9 months

Turkey	C	Letter and personal contact	None	None	Yes	Ministry of Health and Health directory of each province participating	2 months

^*∗*^Sampling procedures codes: A = (almost) entire GP population; B = random national sample (stratified or not); C = random sample in preselected regions; D = mixed procedure (random procedure plus selected GPs); E = “opportunity sampling”/volunteers.

^*∗∗*^The number of reminders was not always well recorded.

^*∗∗∗*^The duration of the field work includes the collection of patient data.

**Table 2:** Participation rates of GPs in a combined GP and patient survey in 35 countries.

Country	GPs invited	GPs participated	Participation rate
Austria	3050	184	6%
Belgium	5000^*∗*^	408	8%
Bulgaria	350	223	64%
Cyprus	90	71	79%
Czech Republic	520	219	42%
Denmark	2000	212	11%
England	1508	171	11%
Estonia	802	137	17%
Finland	1000^*∗*^	288	29%
Germany	3825	238	6%
Greece	300^*∗*^	220	73%
Hungary	400	222	56%
Iceland	95	80	84%
Ireland	2515	169	7%
Italy	Not known	218	Not known
Latvia	545	218	40%
Lithuania	508	225	44%
Luxembourg	120	78	65%
Macedonia	240	143	60%
Malta	78	70	90%
Netherlands	1400	238	17%
Norway	500	198	40%
Poland	665	220	33%
Portugal	800	216	27%
Romania	399	220	55%
Spain	500	428	86%
Slovakia	1000^*∗*^	220	22%
Slovenia	1173	207	18%
Sweden^*∗∗*^	1000	80	8%
Switzerland	2027	199	10%
Turkey	1300	299	23%

^*∗*^Estimate.

^*∗∗*^Reflection of the first wave.

**Table 3:** Assessment of the hypotheses—average (and range) or percentage of the independent variables by three categories of participation rates^*∗*^.

Variable	Low response (*n* = 11) (≤20%)	Middle response (*n* = 9) (21–50%)	High response (*n* = 10) (>50%)
(H1) Average income GPs in €	89,099 (17,500–135,000)	44,160 (10,750–115,000)	48,535 (*n* = 9) (10,800–150,000)
(H2) % with material incentive	82%	56%	30%
(H3) % salaried GPs	9%	33%	40%
(H3) % FFS	18%	0%	30%
(H4) Average survey load	3.2 (1–5)	3.3 (1–5)	1.7 (1–5)
(H5) Average % general trust	44% (*n* = 7) (20–66)	33% (*n* = 8) (21–74)	17% (*n* = 6) (8–29)
(H6) Average number of GPs	14,045 (810–53,790)	7,796 (1,220–7,045)	7,358 (180–39,000)
(H6) Average PC structure	2.29 (2.0–2.5)	2.23 (2.0–2.4)	2.09 (*n* = 9) (1.9–2.4)
(H6) % with support letter professional organization	73%	66%	50%
Average population in millions	19 (1,3–81,7)	17 (2,1–73,7)	10 (0,3–46,7)
Average GDP (EU28 = 100)	118 (47–170)	89 (38–280)	93 (21–310)

^*∗*^No data for Italy.
