# Supplementary material for: General Practitioners' Participation in a Large, Multicountry Combined General Practitioner-Patient Survey: Recruitment Procedures and Participation Rate
Source: Int J Family Med. 2016 Mar 7;2016:4929432. doi: 10.1155/2016/4929432 (PMC4800081; doi:10.1155/2016/4929432)
Supplement: Supplementary file 1 — Additional file 1: A measure of survey pressure was derived from the abstracts submitted to Wonca Europe for its yearly conference in 2008 through 2012. Additional file 2: To assess the representativeness of the responding GPs per country, information on background characteristics of the population of GPs was collected. [file 4929432.f1.docx]

Additional file 1: Survey pressure among GPs by country

A measure of survey pressure was derived from the abstracts submitted to Wonca Europe for its yearly conference in 2008 through 2012. These abstracts are available on <http://www.woncaeurope.org/library/abstracts>, accessed on 6 May 2014 and 8 May 2014. Only abstracts were selected that contained the word ‘questionnaire', in total approximately 1050 abstracts. Within these abstracts only those that reported on a questionnaire among GPs/ FPs were counted. Excluded were surveys among pediatricians and GPs in training/students. This resulted in the identification of 205 surveys/ questionnaires. The numbers per country were grouped into 5 categories: 1=0-1 (n=9 countries), 2=2-4 (n=6), 3=5-6 (n=6), 4=7-10 (n=6) and 5=11 or more (n=4).

| country | Number of surveys | Grouped in 5 categories |
| --- | --- | --- |
| Austria | 6 | 3 |
| Belgium | 5 | 3 |
| Bulgaria | 1 | 1 |
| Cyprus | 0 | 1 |
| Czech Republic | 7 | 4 |
| Denmark | 1 | 1 |
| England/ UK | 6 | 3 |
| Estonia | 5 | 3 |
| Finland | 7 | 4 |
| Germany | 10 | 4 |
| Greece | 4 | 2 |
| Hungary | 2 | 2 |
| Iceland | 1 | 1 |
| Ireland | 6 | 3 |
| Italy | 10 | 4 |
| Latvia | 1 | 1 |
| Lithuania | 2 | 2 |
| Luxembourg | 0 | 1 |
| Macedonia | 1 | 1 |
| Malta | 1 | 1 |
| Netherlands | 25 | 5 |
| Norway | 6 | 3 |
| Poland | 10 | 4 |
| Portugal | 14 | 5 |
| Romania | 2 | 2 |
| Spain | 30 | 5 |
| Slovakia | 3 | 2 |
| Slovenia | 7 | 4 |
| Sweden | 0 | 1 |
| Switzerland | 11 | 5 |
| Turkey | 13 | 5 |
| Europe (countries not named) | 8 |  |

Additional file 2: Age and gender of participating GPs and of the national population of GPs

|  | **National % female GPs** | **QUALICOPC % female GPs** | **National average age** | **QUALICOPC average age** | **Source national statistics** |
| --- | --- | --- | --- | --- | --- |
| Austria | 39.3% | 30.3% | 52.5 | 54.3 | Austrian Chamber of Physicians, Survey 2011 |
| Belgium | 32.0% | 37.6% | 51.4 | 49.2 | website RIZIV, 2012 |
| Bulgaria | NA | 63.2% | NA | 50.5 | NA |
| Cyprus | NA | 49.3% | NA | 47.9 | NA |
| Czech Republic | 61.0% | 69.9% | 53 | 51.8 | Czech Health Statistics, 2010 |
| Denmark | 43.0% | 43.4% | 53.7 | 53.1 | Organisation of General Practitioners in Denmark (PLO), 2012 |
| Estonia | 93.0% | 90.6% | 51.4 | 50.8 | EHIF, 2012; Health Board, 2010 |
| Finland | 63.0% | 71.4% | 44 | 45.0 | FMA, 2012 |
| Germany | 39.5% | 36.1% | 53.28 | 53.9 | National Physician Registry, 2011 |
| Greece | 33.0% | 45.9% | 45 | 43.5 | Greek Statistical Authority 2012 |
| Hungary | 55.0% | 46.9% | 58 | 53.4 | National data, 2011 |
| Iceland | 22.0% | 27.5% | 55 | 54.5 | Dir. of Health, 2011 |
| Ireland | 30.0% | 33.7% | 51 | 50.6 | O’Dowd et a, 2006 |
| Italy | 37.9% | 37.6% | 50.8 | 57.1 | Ministry of Health, 2012 |
| Latvia | 91.0% | 88.5% | 52 | 52.0 | National Health Service, 2011 |
| Lithuania | 85.6% | 88.4% | 49.6 | 51.2 | Ministry of Health, 2011 |
| Luxembourg | 33.0% | 36.8% | 52 | 49.1 | Health ministry, 2010 |
| FYR Macedonia | 70.0% | 83.9% | 49 | 45.7 | Health Insurance Fund, 2013; Macedonian Association of GP/FM Specialists, 2013 |
| Malta | 26.0% | 29.0% | NA | 46.8 | Health Care Professions Act Medical Council Family Medicine Specialist Register, 2012 |
| the Netherlands | 39.9% | 28.1% | 48.5 | 53.1 | NIVEL, 2012 |
| Norway | 38.0% | 39.1% | 48.5 | 45.7 | Statistics Norway, 2011/2 |
| Poland | NA | 63.6% | NA | 49.5 | NA |
| Portugal | NA | 60.5% | NA | 51.4 | NA |
| Romania | 75.9% | 83.2% | NA | 52.0 | NSI, 2011 |
| Slovakia | 68.4% | 67.9% | 54.8 | 52.6 | Štatistická ročenka (Statistical yearbook) MZ SR, 2010 |
| Slovenia | 58.9% | 75.4% | 47.3 | 48.9 | Medical Chamber of Slovenia, 2011 |
| Spain | 48.4% | 63.2% | 43.11 | 49.7 | Ministry of Health, 2011 |
| Sweden | NA | 54.6% | NA | 52.0 |  |
| Switzerland | 26.5% | 22.1% | 53.4 | 55.0 | FMH, 2012; Swiss association of FPs, 2012 |
| Turkey | NA | 30.5% | NA | 44.0 |  |
| UK | 46.0% | 37.9% | 42,4 | 46.6 | BMA, 2012 |

NA= Not Available
